# Severe Hyponatremia due to Levofloxacin Treatment for* Pseudomonas aeruginosa* Community-Acquired Pneumonia in a Patient with Oropharyngeal Cancer

**DOI:** 10.1155/2016/5434230

**Published:** 2016-10-25

**Authors:** Mihaela Mocan, Sorin Nicu Blaga

**Affiliations:** ^1^Department of Internal Medicine, University of Medicine and Pharmacy, Cluj-Napoca, Romania; ^2^1st Medical Clinic, County Emergency Hospital, Cluj-Napoca, Romania

## Abstract

Hyponatremia (serum Na levels of <135 mEq/L) is the most common electrolyte imbalance encountered in clinical practice, affecting up to 15–28% of hospitalized patients. This case report refers to a middle-aged man with severe hyponatremia due to Syndrome of Inappropriate Antidiuretic Hormone Secretion related to four possible etiological factors: glossopharyngeal squamous cell carcinoma, cisplatin treatment, right basal pneumonia with* Pseudomonas aeruginosa*, and the treatment with Levofloxacin. This case report discusses a rare complication of common conditions and of a common treatment. To our knowledge this is the first case of hyponatremia related to Levofloxacin and the second related to fluoroquinolones.

## 1. Introduction

Hyponatremia (serum Na levels of <135 mEq/L) is the most common electrolyte imbalance encountered in clinical practice, affecting up to 15–28% of hospitalized patients. Both moderate and especially severe hyponatremia (Na <125 mEq/L) found in newly admitted hospital patients are linked with a significantly elevated in-hospital mortality of 28% compared to 9% in-hospital mortality in normonatremic, matched controls [1].

Despite the frequency of this condition, the etiologic diagnosis and the management of hyponatremia are neither easy nor optimal [2]. This may be attributable to the diversity of underlying disease states associated with the condition and, until the last few years, a lack of targeted treatments.

## 2. Case Presentation

A 59-year-old male was brought in to the emergency department of Emergency County Hospital of Cluj-Napoca presenting with dizziness, psychomotor agitation, delirium with visual and auditory hallucinations, and temporal and spatial disorientation. The patient was known for chronic tobacco use (40 cigarettes/day) and heavy alcohol consumption.

History taking revealed that his symptoms had started 1 month previously when he was hospitalized in the Pneumology Unit suffering from right chest pain, dyspnea, fatigue, productive cough, 38°C fever, and shaking chills. He was diagnosed with* Pseudomonas aeruginosa* right basal pneumonia based upon findings on physical examination and paraclinical explorations (chest CT scan, blood cultures, bronchoscopy with cytology, and aspirate cultures). Consequently, antibiotic treatment was initiated, at first, ceftriaxone 2 g/day (7 days long), but as there were no clinical signs of resolution, the treatment was switched to amikacin and colistin, for the following 10 days. On admission to our service he was still on antibiotic treatment for pneumonia, that time with Levofloxacin 500 mg per day, which had been started 4 days previously.

His medical history included a glossopharyngeal squamous cell carcinoma cT3N3Mx treated with combined chemotherapy (docetaxel, cisplatin, and capecitabine) and radiation therapy.

The clinical examination, at the admission time, revealed an overweight patient (BMI = 27 kg/m^2^) with warm and moist skin and psychomotor agitation. Cerebral CT scan was performed in the Emergency Room and it did not show any focal masses or other pathological findings that could explain the acute onset of the neurologic manifestations.

The lab exams (Table 1) showed a low serum sodium concentration of 114 mEq/L indicating severe hyponatremia, with correspondingly low serum osmolality of 233.9 mOsm/kg, normal creatinine, urea, and uric acid. His urinary Na was high 40 mEq/L and his central venous pressure was normal (5 cmH_2_O). Given the severity of the hyponatremia a treatment with hypertonic saline 3% was started in the Emergency Room and his existing Levofloxacin treatment was stopped. He was admitted to the Internal Medicine Department for surveillance. During the following 24 h the patient serum Na rose to 120 mEq/L (6 mEq/L). Hypertonic saline treatment was stopped and replaced with fluid restriction (800 mL/day), as the patient did not meet the exclusion criteria for applying this treatment (mentioned above). Within the next 72 h serum Na increased to 131 mEq/L and his symptoms subsided. Adrenal insufficiency and severe hypothyroidism were excluded through laboratory tests. Therefore, the etiology of euvolemic hypotonic hyponatremia diagnosed in our patients was likely to be Syndrome of Inappropriate Antidiuretic Hormone Secretion (SIADH) fulfilling both essential and supplemental criteria for the diagnosis [3].

**Figure 1:**
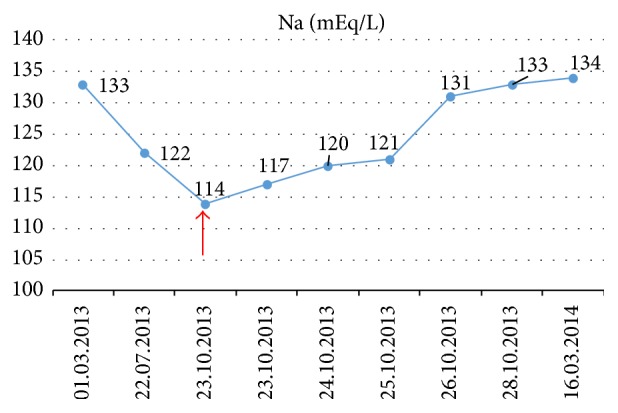
Evolution of serum Na levels since the diagnosis and treatment (03/01/2013) of glossopharyngeal squamous cell carcinoma until six months after discharge from Internal Medicine Department (03/16/2014). The moment of hospital admission is marked with the red arrow (10/23/2013).

Looking back into his past medical file, as shown in Figure 1, we discovered that our patient had been hyponatremic ever since diagnosed with neck cancer. After chemotherapy the Na levels decreased slowly by 10 mEq/L, the patient being asymptomatic. During the acute episode of pneumonia and Levofloxacin treatment, hyponatremia suddenly aggravated and the patient became symptomatic. The mild fluid restriction (maximum 1500 mL/day), normal Na intake, and the avoidance of any medication that could affect sodium levels allowed the maintenance of Na concentration close to physiological limit, for more than 6 month after discharge (Figure 1).

## 3. Discussions

In our case, we identified at least 4 possible causes of SIADH and hyponatremia:

- Glossopharyngeal squamous cell carcinoma
- Cisplatin treatment
- Right basal pneumonia with* Pseudomonas aeruginosa*
- The treatment with Levofloxacin

Each of these etiological factors will be discussed separately beneath.

Hyponatremia is common in malignant solid tumors (up to 25% of all patients) either as part of the underlying disease or due to drug side effects [4]. SIADH has been reported in >3% of patients with head and neck cancer, most often in patients with lesions in the oral cavity and less frequently in those with lesions in the larynx, nasopharynx, hypopharynx, or other sites [5], frequently due to ectopic secretion of antidiuretic hormone (ADH). Treatment of lung cancer with chemotherapeutic agents such as platinum derivatives or vinca alkaloids may lead to hyponatremia. The development of hyponatremia, during cisplatin therapy, bears special mention for our case. Cisplatin stimulates ADH secretion to cause SIADH, but it can also directly damage renal tubules to interfere with sodium reabsorption, which in rare cases may lead to hyponatremia via salt wasting nephropathy [4].

Besides malignancies, SIADH may be caused by a variety of other conditions, including CNS disorders, pulmonary disorders (e.g., tuberculosis, pneumonia, and acute respiratory failure), HIV infection, prolonged strenuous exercise, and drugs [3].

A recent German study reported a high incidence of hyponatremia (31.8%) among patients with community-acquired pneumonia [6]. The degree of hyponatremia severity seemed to correlate with the patients' comorbidities (such as chronic heart failure, chronic renal disease, diabetes mellitus, and malignancies), higher severity of pneumonia, and higher inflammatory biomarkers. However, the association of these comorbidities with sodium levels was weak and disappeared after inclusion in a multivariate model. The relationship of hyponatremia and higher pneumonia severity probably reflects the presence of hypovolemia, severe sepsis, and subsequent activation of vasopressin and natriuretic peptides secretion [6]. The decrease in the ability to reduce urine osmolality and excrete water loads and the increasing levels of ADH in the absence of antibiotic treatment [7] might be incriminated in pneumonia-induced hyponatremia.

As for the antibiotic treatment, it seems that Levofloxacin could be a cause for SIADH. There is no data in the literature regarding hyponatremia to Levofloxacin, but there are case series presentations of quinolones' side effect. Fluoroquinolones have the potential to cause SIADH. The likely mechanism of hyponatremia is that quinolones cross the blood-brain barrier and stimulate the *γ*-aminobutyric acid and N-methyl-D-aspartate receptors, which leads to the synthesis and release of antidiuretic hormone [8, 9]. An objective causality assessment using the Naranjo scale could have been useful to demonstrate the link between Levofloxacin use and hyponatremia.

Finally, the link between alcohol ingestion and hyponatremia is worth mentioning. Hyponatremia may be one of the laboratory signs of chronic alcohol abuse (up to 17% of the alcoholics), along with frequent presentation to ER department for recurrent unexplained falls, poorly controlled hypertension, and/or gastrointestinal symptoms [10]. The mechanisms for hyponatremia in alcoholics include hypovolemia, pseudohyponatremia due to alcohol-induced hypertriglyceridemia, beer potomania syndrome, and rarely SIADH or cerebral salt wasting. In our case, heteroanamnesis revealed the stop of alcohol ingestion 1 year prior to presentation, so chronic alcoholism was not considered as a cause of hyponatremia [10, 11]. However, a CNS disorder associated with alcohol consumption (Wernicke encephalopathy or Korsakoff dementia) was initially suspected to be the cause of the delirium at presentation, so high-dose thiamine was administered intravenously for 5 days.

Apart from the fact that persistent hyponatremia may affect the patients' quality of life, it seems to be a negative prognostic marker for overall survival. Large studies including patients with malignant disease demonstrate that serum Na level <130 mEq/L is independently associated with a 2–2.5-fold greater risk for in-hospital mortality [12].

## 4. Conclusions

In summary, we presented a case of hyponatremia of multifactorial etiology that was promptly investigated and corrected. To our knowledge this is the first case of hyponatremia related to Levofloxacin and the second related to fluoroquinolones. The patients' outcome at six months was a good one, bearing in mind the comorbidities.

## Figures and Tables

**Table 1:** Laboratory results at admission and in evolution.

Analysis	At admission	During hospitalization	At discharge	Normal range
Sodium (mEq/L)	***114***	***131***	***133***	135–145
Sodium deficiency (mEq)	**284.4**	—	—	—
Potassium (mEq/L)	3.8	4.1	4	3.5–5.1
Chloride (mEq/L)	98	102	104	96–106
Urea (mg/dL)	24	30	25	15–45
Creatinine (mg/dL)	0.58	0.72	0.80	0.57–1.12
Uric acid (mg/dL)	2.9	3	3.5	2.6–7
Osmolality (mOsm/kg)	233	—	285	285–295
Glycemia (mg/dL)	89	90	85	70–110
TSH (mIU/mL)	3.15	—	—	0.7–4.2
Cortisol (nmol/L)	327	—	—	171–536
Total protein (g/L)	6.5	—	—	6.4–8.3
WBC (×10^3^/mm^3^)	4.7	—	5	4–10.5
Hemoglobin (g/dL) Hematocrit (%) Platelet (×10^3^/mm^3^)	8.3 24.1 344	— — —	8.5 25 350	11.5–15.5 36–48 150–400

Urine chemistry				
Osmolality (mOsm/kg)	116	450	800	500–800
Na (mEq/L)	40	60	130	40–220
K (mEq/L)	25	56	60	20–125
Density	1025	1030	1031	1015–1025
Volume status: PVC (cmH_2_O)	5	—	—	—

Na deficiency = [120-Na_seric_  (mEq/L)] × *G*  (kg) × 0.6.
